# Equitable Allocation of Antiretrovirals in Resource-Constrained Countries

**DOI:** 10.1371/journal.pmed.0020057

**Published:** 2005-02-22

Antiretroviral drugs change the lives of patients with HIV/AIDS—if they have access to them. Most patients in resource-poor countries cannot afford the drugs. Major initiatives are under way to expand access to antiretrovirals in developing countries, but the number of individuals in need of the drugs currently vastly exceeds the supply, and will continue to do so for the foreseeable future. These circumstances make for difficult decisions about treatment allocation. David Wilson and Sally Blower have shown how it is possible to design an equitable antiretroviral allocation strategy, that is, to come up with a plan that would give each individual with HIV an equal chance of receiving antiretrovirals. Their novel spatial model enables them to model the “spatial diffusion” of antiretrovirals in a resource-constrained country.[Fig pmed-0020057-g001]

**Figure pmed-0020057-g001:**
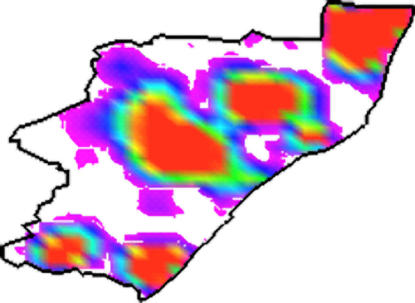
Modeling allocation of antiretrovirals in KwaZulu–Natal

Based on the premise that only a limited number of drugs will be available and only a limited number of health-care facilities can be used for drug distribution (each of them serving the population in a specific area), they determine an optimal equitable allocation strategy. They then apply this approach to a practical example—the equitable allocation of antiretrovirals to patients with HIV/AIDS in the South African province of KwaZulu–Natal. Using data from a detailed rollout plan for antiretrovirals designed by the South African government, they come up with an allocation strategy that differs substantially from the current governmental plan for the province.

KwaZulu–Natal has a total of 54 health-care facilities, of which 17 are assigned to allocate antiretrovirals under the current plan. It is the largest province in South Africa, with a population of about 9.4 million, and it has more people with HIV than any other province (about 21% of all cases in South Africa). Wilson and Blower assume that the available amount of antiretrovirals can treat 10% of the individuals with HIV in KwaZulu–Natal. Modeling the 17 health-care facilities and the 51 communities of individuals with HIV, they determine the amount of drugs to allocate to each facility to achieve equitable access by patients throughout the province. They then extend the analysis assuming that additional health-care facilities could be made available to distribute drugs. They conclude that in order to achieve the greatest degree of treatment equality, all 54 health-care facilities should be used, and they should, on average, each serve the population within a radius of 50 km.

Wilson and Blower discuss how their model can be adjusted and therefore used by policy makers in resource-constrained countries to determine a scientifically based allocation strategy for limited resources based on a number of specific objectives. They also recognize that there are other considerations that influence ethical treatment allocation besides equity, for example, the desire to maximize epidemic reduction, or the imperative to give priority to the least advantaged individuals They believe that their model can be adjusted and therefore “used by policy makers to determine an optimal scientifically based allocation strategy” for a number of specific objectives. Another possibility would be to apply the equity strategy to allocate drugs to particular health-care facilities (thereby achieving equality in accessibility), and then take additional ethical considerations into account at the community level.

